# Supplementary material for: Deterministic Assessment of the Risk of Phthalate Esters in Sediments of U-Tapao Canal, Southern Thailand
Source: Toxics. 2020 Oct 26;8(4):93. doi: 10.3390/toxics8040093 (PMC7712367; doi:10.3390/toxics8040093)
Supplement: Supplementary file 1 [file toxics-08-00093-s001.pdf]

toxics

Deterministic Assessment of the Risk of Phthalate Esters in Sediments of U-Tapao Canal, Southern Thailand

Okpara Kingsley 1,2,* Banchong Witthayawirasak 1,2

Risk Quotient (RQ) Method

The Ecological risk assessments of PAEs was also carried out by using the risk quotient method (RQ) in accordance to the European Commission's Technical Guidance Document [21, 22]. RQ for detected PAEs congeners was estimated by dividing the measured environmental concentration (MEC) with the predicted no-effect concentration (PENC) as indicated in equation 4. In this study, the mean and highest MEC values were used for calculating general RQmean and worst-case scenarios RQmax, respectively. The risk quotient of the mixture (RQ_{mix}) of PAEs was calculated based on the summation of individual RQ values of PAEs congeners detected in sediments as indicted in the text. The criteria used to assess the ecological risk assessment were RQ > 1 (the log Kow of PAE congener between 3 and 5) showed high risk whereas RQ > 10 (log Kow > 5) suggested high risk [22, 29]. The US EPA ECOTOX database provided the chronic or acute toxicity data of PAEs to the algae, crustaceans, and fish for this study (http://cfpub.epa.gov/ecotox/). PNEC values in the sediment used in this study are indicated in Table below.

Table S1. Toxicity data of 3 PAEs to sensitive surrogate aquatic species.

PAEs	Population	Species	Toxicity data	AF	$PNEC_{water}$	$PNEC_{sediment}$
DBP	Algae	Pseudokirchneriella subcapitata	96 h, population, NOEC = 210	10	21	2.33
	crustacean	Americamysis bahia	21 d, mortality, NOEC = 260	10	26	2.88
	Fish	Oncorhynchus mykiss	99 d, growth, NOEC = 100	10	10	1.11
DEHP	Algae	Pseudokirchneriella subcapitata	96 h, population, EC50 = 100	1000	0.1	3.25
	Crustacean	Mytilus edulis	21 d, mortality, NOEC = 42	50	0.84	13.6
	Fish	Gasterosteus aculeatus	28 d, mortality, NOEC = 300	50	6	32.5
DiNP		Daphnia magna	21 d, mortality, NOEC = 34	100	0.34	181.4

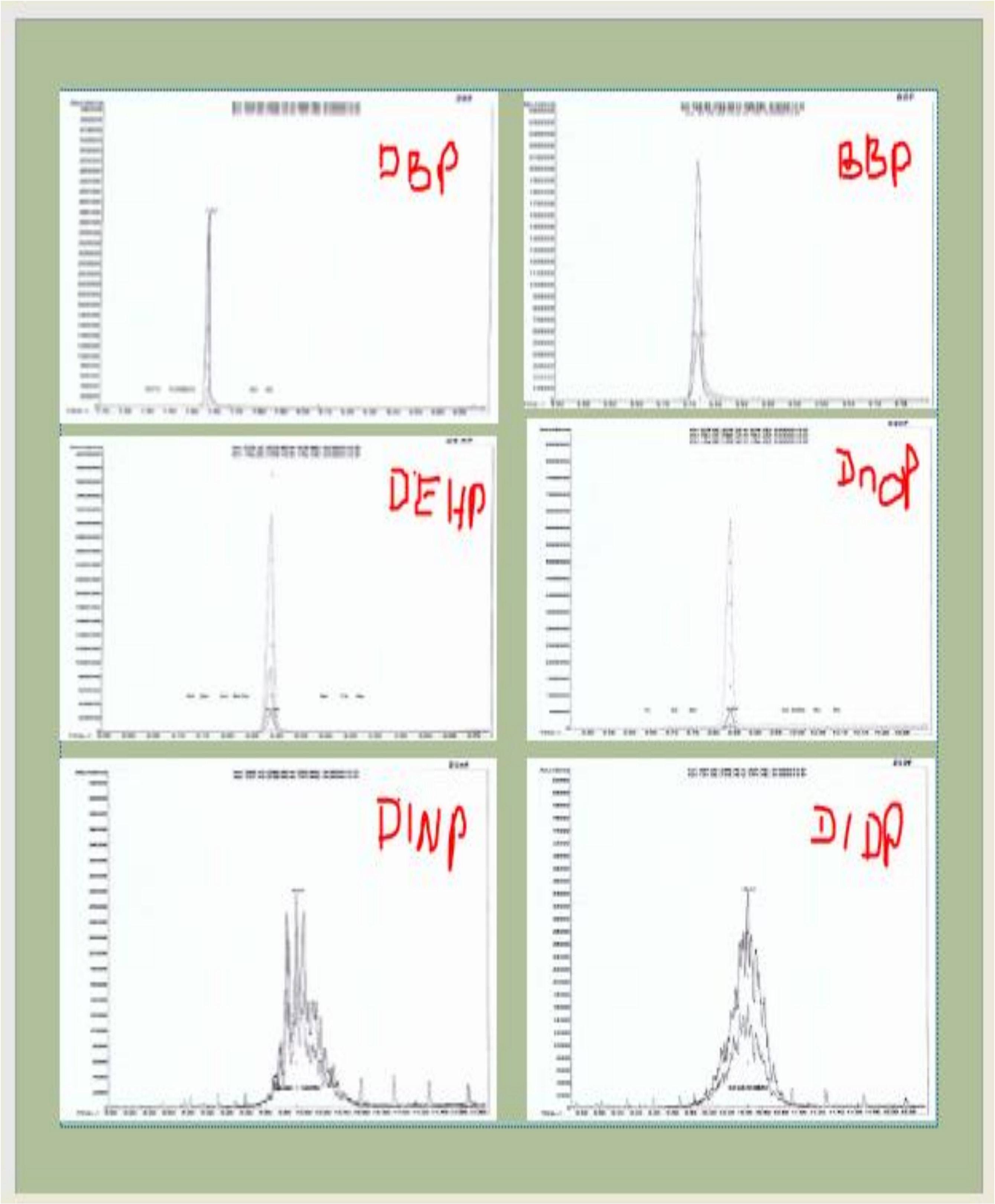

Figure S1. of DBP, BBP, DEHP, DnOP, DiNP and DIDP.

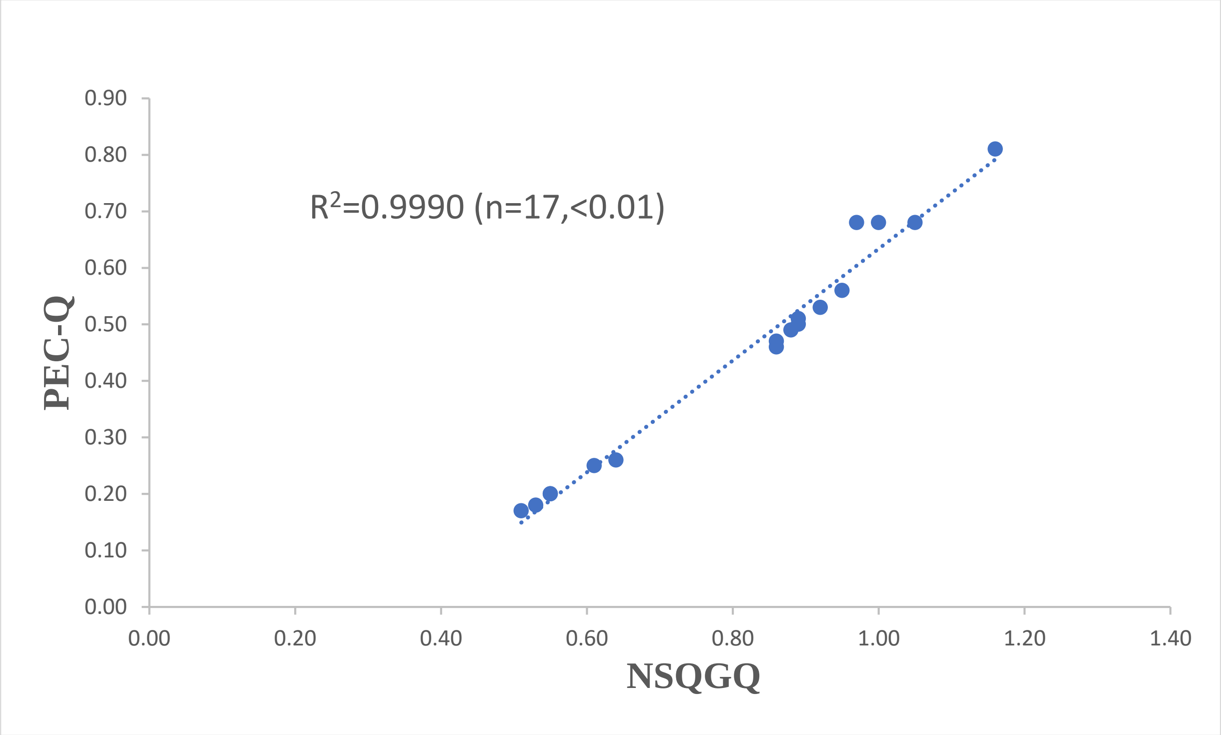

Figure S1 Linear relationship between the PEC-Q and NSQGQ.

Toxics **2020**, *8*, x FOR PEER REVIEW 3 of 3
